# Myeloid Extracellular Vesicles: Messengers from the Demented Brain

**DOI:** 10.3389/fimmu.2016.00017

**Published:** 2016-01-29

**Authors:** Annamaria Nigro, Federico Colombo, Giacomo Casella, Annamaria Finardi, Claudia Verderio, Roberto Furlan

**Affiliations:** ^1^Division of Neuroscience, Institute of Experimental Neurology, San Raffaele Scientific Institute, Milano, Italy; ^2^CNR Institute of Neuroscience, Milano, Italy; ^3^IRCCS Humanitas, Rozzano, Italy

**Keywords:** extracellular vesicles, neuroinflammation, neurodegeneration, Alzheimer disease, multiple sclerosis

## Abstract

Blood-borne monocyte derived cells play a pivotal, initially unrecognized, role in most central nervous system disorders, including diseases initially classified as purely neurodegenerative (i.e., Alzheimer’s disease, Parkinson’s disease, and ALS). Their trafficking to the brain and spinal cord has been extensively studied in classical neuroinflammatory disorders such as multiple sclerosis. Central nervous system resident myeloid cells, namely microglia and perivascular macrophages, also are in the spotlight of investigations on neurological disorders. Myeloid cells, such as infiltrating macrophages and microglia, have been described as having both protective and destructive features in neurological disorders, thus identification of their functional phenotype during disease evolution would be of paramount importance. Extracellular vesicles, namely exosomes and shed vesicles, are released by virtually any cell type and can be detected and identified in terms of cell origin in biological fluids. They therefore constitute an ideal tool to access information on cells residing in an inaccessible site such as the brain. We will review here available information on extracellular vesicles detection in neurological disorders with special emphasis on neurodegenerative diseases.

## Introduction

Phagocytes, and in particular macrophages and microglia, are in the spotlight of investigations on the pathogenesis of neurodegeneration, involving disorders such as the progressive phase of multiple sclerosis (MS) and more classical neurodegenerative diseases such as Alzheimer’s and Parkinson’s disease (AD and PD). While in all these cases, classical anti-inflammatory therapies have failed, and there is no doubt that an inflammatory component is always present. The hypothesis that malfunctioning or dysregulated myeloid cells are the cause of chronic inflammation associated to neurodegeneration also derives from the knowledge that the innate immune system is highly plastic. In this respect, it may control the initiation of the inflammatory process as well as transition from a purely destructive process to termination of inflammation and tissue reconstruction. Data available in the literature do not compose a coherent picture for several reasons. Inflammation is clearly a protective process, and it is difficult to identify and distinguish the altered component, if present. Second, there are numerous different myeloid cells in the brain, such as microglia, infiltrating and perivascular macrophages and dendritic cells. All are very plastic in their functional phenotype and endowed with peculiar abilities, playing different roles during neuroinflammation. This confusing picture has lead to the common use of an oversimplified definition of these functional phenotypes (M1 = pro-inflammatory; M2 = anti-inflammatory and pro-reparative) and to a superficial definition of their role in the pathogenesis of neurodegeneration (M1 = detrimental; M2 = protective). Data in experimental models, however, are not in accordance with this straightforward working hypothesis, and the M1/M2 paradigm is no longer the consensus in the field, knowing that the myeloid cell phenotype *in vivo* is very complex. Data in the human setting are, however, very difficult to obtain since there is currently no way to investigate the functional phenotype of CNS-resident myeloid cells during disease development. Taking AD as a prototypical example, we will try in the present review to suggest a possible line of investigation able to contribute novel data on the role of myeloid cells during neurological disorders.

## Myeloid Cells Phenotype in AD

While for decennia, the focus of research in AD had been almost exclusively on the mechanisms leading to extracellular accumulation of the amyloid β peptide 1–42 (Aβ_1–42_) and the deposit of neurofibrillary tangles (NFTs), more recently several investigators have explored the role of the immune system in AD. The inflammatory response associated with AD is primarily driven by the resident cells of the brain such as microglia, perivascular macrophages, and reactive astrocytes and by non-CNS resident cells like monocytes/macrophages. It is not very clear when inflammatory response starts in AD. It was originally assumed that neuroinflammation occurs only at late stages of AD and that glial cell activation accompanies but does not significantly contribute to amyloid pathology (1, 2). More recent data, mostly from the debated genetic mouse models of AD, indicate that the involvement may be more precocious (3). It appears reasonable to think that microglia is the first myeloid cell to be activated during AD, but the CNS has a rich complement of non-microglial myeloid cells including meningeal and choroid plexus macrophages, as well as perivascular macrophages. Of these, perivascular macrophages seem to have a particularly crucial role in the physiological removal of Aβ_1–42_ and protection from amyloid pathology (4) and are continuously replaced by circulating monocytes. Both macrophages and microglia cells can be polarized into M1 and M2. This activation is dynamic and plastic and built up in response to the changes of the microenvironment. Phenotypic classification of macrophages is further hampered by the lack of exclusivity of some of the classical M1 and M2 marker, and by the difficulty to overlap it with functional classification (inflammatory vs. wound healing vs. regulatory macrophages) (5). The identification of new phenotypic markers may allow to overcome these difficulties in the near future (6, 7). Using conventional markers, both M1 and M2 phenotypes co-exist during both early and late AD (3), possibly linked to slightly different disease phenotypes (8). The very lively debate on the role of myeloid cells during neurodegeneration is caused by the uncertainties on their actual activation phenotype during disease development. This is due to the lack of handy investigation tools, such as appropriate biomarkers, to assess microglia activation *in vivo*, with the notable exception of PET imaging using the tracer [11C] (R)-PK11195 (9). The latter, however has low resolution and does not provide any information on the myeloid cell and activation phenotype.

## Extracellular Vesicles to Investigate Microglia

In the experimental mouse model for multiple sclerosis (MS) and in human MS patients, we described that microglia release microvesicles that can be detected in the CSF and that can contribute to disease pathogenesis by delivering a pro-inflammatory signal (10).

The recent discovery that cell-derived extracellular microvesicles (EVs) are not artifactual *in vitro* entities, as considered for many years, has somehow revolutionized the field of cell-to-cell communication. There is a growing interest of researchers for microvesicles, and it seems that there is no field of biomedicine where they are not investigated.

Circulating EVs contribute to the development of cancer, autoimmune, and cardiovascular diseases, and the elevated number of shedding vesicles in plasma and/or other biological fluids (CSF, saliva, amniotic fluid, synovial fluid, breast milk, and urine) (11) correlates with the acute or active phase of many diseases (tumor progression, tissue destruction, etc.), suggesting that they can be used as potential predictive and diagnostic biomarkers for a large number of pathologies.

**Figure 1:**
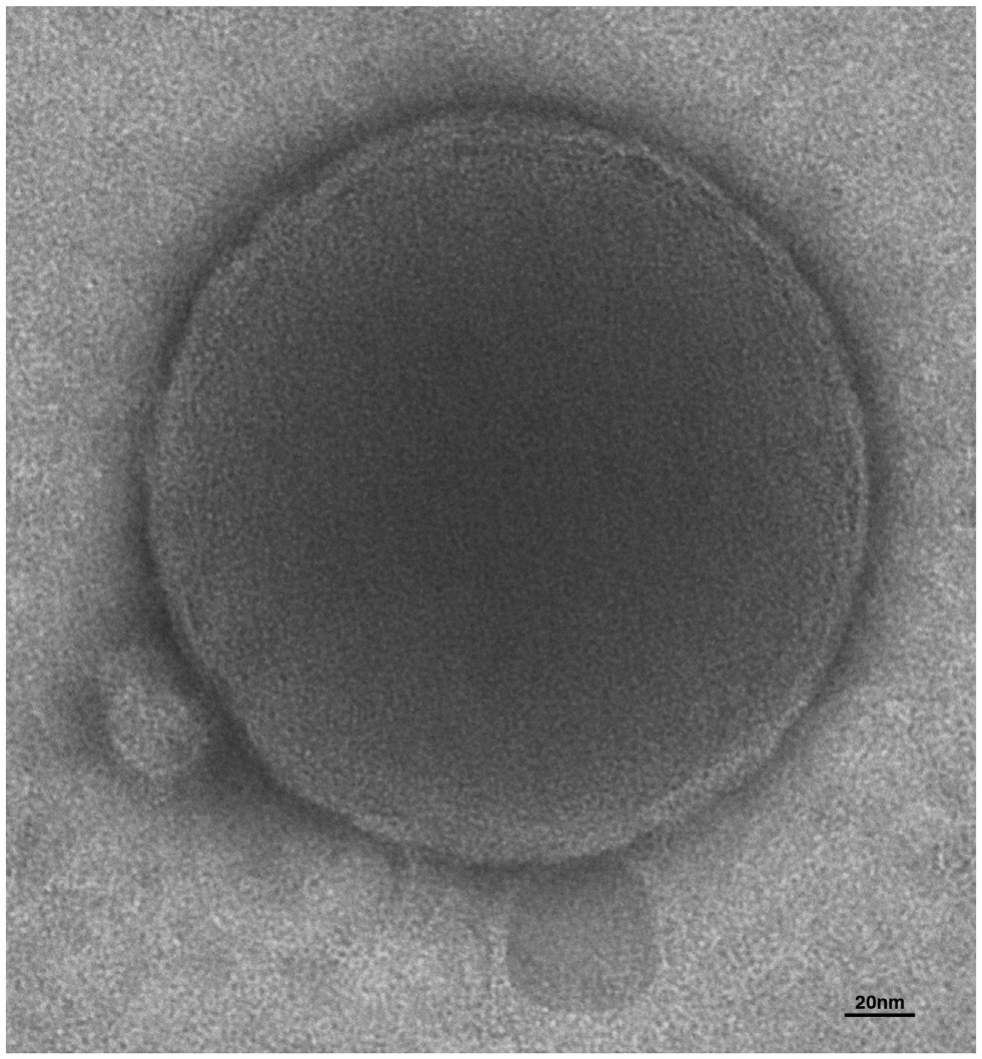
**Electron microscopy of extracellular vesicles**. The human microglia cell line, CHME-5 was stimulated with ATP to induce release of EVs. Supernatant was analyzed by transmission electron microscopy. Integrity, round shape, and lipid bilayer of the membrane (approximately 7 nm) can be appreciated from the picture of this shed vesicle of about 250 nm of diameter.

So far, two main EVs types have been isolated and described according to their different size and density: shedding vesicles and exosomes. Shedding vesicles are 100–1000 nm sized vesicles derived from the cell plasma membrane upon surface bending and budding as a consequence of lipid scrambling and cytoskeleton remodeling (Figure 1). These deep but reversible structural changes are the final step of a complex signaling network that has not been clarified yet. Shedding vesicles biogenesis is strongly enhanced by extracellular ATP and its structural analogs (1) as well as by some other few “unphysiological” triggers such as calcium ionophores or phorbolester derivatives (12) whose overall effect consists in rising the cytosolic calcium levels. On the other hand, exosomes are nanovesicles (20–100 nm) of endosomal origin, as they are generated by the invagination of the late endosomes membrane. Resulting organelles have been defined as multivesicular bodies whose intraluminal vesicles are the exosomes. The exocytosis of multivesicular bodies eventually culminates in the release of exosomes outside the cell. The interest and excitement for EVs has been sudden and exponential, leading to confusion in the nomenclature and classification of microparticles necessitating urgent revision for the sake of clarity in the field (13, 14). The molecular composition of shedding vesicles and exosomes is highly heterogeneous, but similar to the cells of origin. Their content includes proteins (signaling molecules, receptors, integrins, and cytokines), bioactive lipids, nucleic acids (miRNA, mRNA, and DNA), and organelles (15) through which they can influence recipient cells (11).

This influence is relevant in physiology, but of course also during pathology. Particularly relevant is the influence on the immune system, the system devoted to protect tissues from insults and to foster regenerative processes. Extracellular vesicles are involved in all immune activities, and thus their effect in neurological disorders can be protective or detrimental, as the influence of the immune system itself (16).

## Extracellular Vesicles in Alzheimer’s Disease

Multiple lines of research indicate that extracellular vesicles are involved in the pathogenesis of AD. To date, the potential role of EVs in AD is object of debate and evidences for either beneficial or detrimental action have been reported (17–26).

Yuyama and co-workers have recently suggested a neuroprotective role of EVs in AD describing a novel function for exosomes as scavengers of neurotoxic Aβ (21). In particular, addition of an exosome/Aβ mixture to primary cortical cells significantly suppressed the formation of toxic oligomers and neuronal toxicity revealing the capability of exosomes to trap Aβ and to promote its clearance by microglia (27). Additionally, Aβ pathology and amyloid deposition diminished following intracerebral infusion of exosomes isolated from cultured neuroblastoma cells in a mouse model of AD (20). These findings suggest a coordinated mechanism by which neurons and neighboring microglia use exosomes to clear Aβ_1–42_. Authors proposed to use exosomes as a novel therapeutic approach to prevent plaque deposition in AD (21). The potential neuroprotective role of exosomes in AD is consistent with a recent study on a mouse model of familial AD. In this study, the presence of presenilin-2 mutations is associated with reduced ability of neurons to eliminate cystatin C, APP, and their toxic metabolites trough exosomes (25). Recent reports focused on role of neprilysin in AD pathology, supporting a scenario in which neprilysin-loaded exosomes contribute to Aβ clearance in the brain. Accordingly, a recent study demonstrated for the first time that adipose tissue-derived mesenchymal stem cells produce neprilysin-bound exosomes. Co-culture experiments indicated that mesenchymal stem cells-derived exosomes contributed to decrease of the secreted Aβ levels in N2a cells, suggesting a therapeutic potential of microvesicle-bound neprilysin for AD treatment (28).

On the other hand, the neurotoxic role of EVs was initially proposed after the observation that Aβ species involved in AD can be encapsulated into neuronal exosomes to be released in the extracellular space. Aβ_1–42_ is generated in early endosomes, sorted to multivesicular bodies in APP-expressing neuroblastoma cells, and released via exosomes after fusion of multivesicular bodies with the plasma membrane into the extracellular environment (29, 30). The identification of exosome-associated proteins, such as Alix and flotillins, in amyloid plaques in the brains of mice (31) and post-mortem human AD patients (29) supports a role for exosomes in spreading Aβ_1–42_ aggregates to these sites during disease progression and represents a novel mode of amyloid transmission (32). Further evidence of a potential role of exosomes in AD comes from recent studies demonstrating that also proteins and peptides, such as APP, APP C-terminal fragments, APP intracellular domain, associated with AD, can be selectively released in association with exosomes and can contribute to the pathology (29, 30, 33).

The neurotoxic role of EVs in AD is also sustained by data showing the increase of microglial EVs production in patients with neurodegenerative diseases (17, 18). Recently, our group demonstrated that the increase of EVs derived from myeloid cells (IB4^+^) measured in CSF of AD and mild cognitive impairment (MCI) patients correlates with classical biomarkers of neuronal injury (i.e., t-Tau, p-Tau, and Aβ_1–42_), demonstrating the diagnostic potential of EVs (18). Notably, CSF EVs levels were positively associated with white matter damage in MCI patients and hippocampal atrophy in AD patients, as detected by MRI (18). Accordingly, high levels of p-Tau associated with exosome have been found in blood of patients with prodromal AD (34). Moreover, EVs released by microglia exposed to Aβ_1–42_ *in vitro* promote formation of soluble neurotoxic Aβ_1–42_ species from extracellular insoluble aggregates in neuron cultures thereby acting as potent drivers of neuronal damage (17). The observation that microglia release Aβ species in association with EVs, together with the evidence that activated microglia constantly surround amyloid deposits (35) suggests the existence of a potential mechanism of cell–cell communication by which EVs spread Aβ pathology through the brain. EVs seem to be involved also in the diffusion of aggregated Tau during AD (36, 37). In a recent study, Saman and co-workers (36) show that CSF exosomes from AD patients contain hyperphosphorylated oligomeric tau secreted by neurons, suggesting that exosome-mediated secretion of phosphorylated tau may play a significant role in the abnormal processing of tau and in the genesis of elevated CSF tau in early AD. Notably, microglia may also facilitate tau protein propagation to neurons by phagocyting and secreting tau protein in exosomes (38). Nevertheless, the role of EVs in protein-aggregation diseases requires further investigations (24).

**Figure 2:**
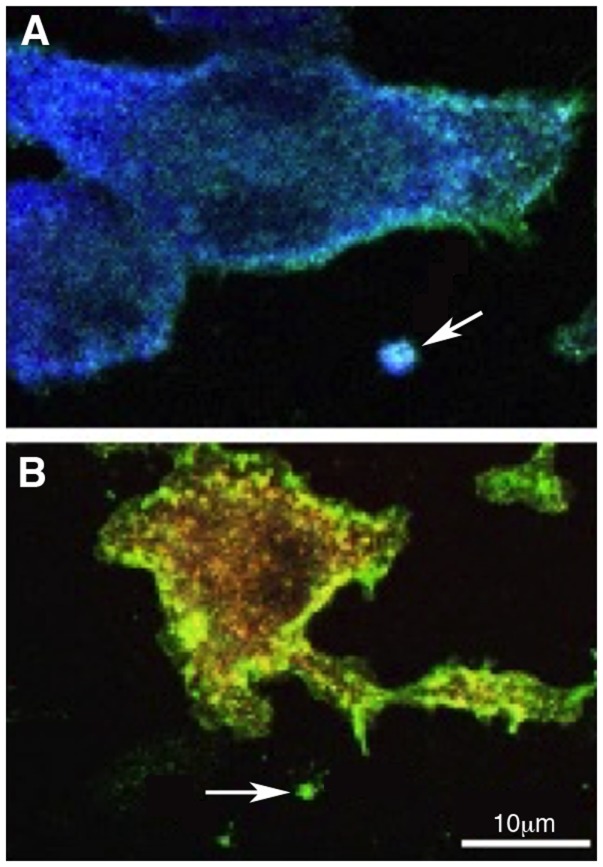
**Polarized myeloid cells release polarized microvesicles**. Murine macrophages were polarized in the presence of IFN-γ [M1 **(A)**] or IL-4 [M2 **(B)**] and stained with anti-IB4 (green), anti-Iba-1 (blue), anti-CD206 (red), and DAPI (gray). IB4 stains all myeloid cells, Iba-1 is an M1 marker and is up-regulated in **(A)** while CD206, an M2 marker, is up-regulated in **(B)**. Arrows in **(A,B)** indicate putative shed vesicles (of approximately 1 μm of diameter) displaying polarization markers similar to their.

Overall, increased levels of EVs in the CSF, together with altered value of soluble proteins in AD patients, may represent an emerging strategy for the identification of possible predictive biomarkers and/or therapeutic targets for AD. There is no doubt, however, that additional information may be gained by analyzing the EVs content. While, in fact, available data link the release of EVs in the CSF to microglia activation, we still do not have a tool to investigate the functional phenotype of activated microglia. In particular, toxic or protective role of EVs in AD may be linked to the functional phenotype of their parent cells. Since EVs represent, in their content, the cell of origin, microglia microvesicles are potentially the source of this information. Our group provided proof of principle for myeloid cells showing how M1 and M2 markers in microvesicles allow the identification of the phenotype of parental macrophages, *in vitro* and *in vivo* (Figure 2) (39).

## Conclusion

There is a growing interest in myeloid cells in neurodegenerative disorders such as the progressive forms of MS, AD, PD, and ALS. In general terms, this originates from the presence of an immune/inflammatory signature in all of these diseases on one side and on the failure of classical anti-inflammatory or even immune-suppressive treatments on the other. CNS-resident and CNS-infiltrating myeloid cells are able to modify their phenotype according to the environment in a matter of hours and their location behind the blood brain barrier makes the scarcely accessible to current treatments. The variety of activation patterns and stimuli of these cells and the lack of knowledge on their involvement in neurodegeneration leaves open the door to all sorts of speculations on their protective rather than pathogenic role in neurodegeneration. Investigating their biology and their behavior would be possible in experimental mouse models, but both the model for progressive MS and those for neurodegenerative diseases, such as AD, are scarcely representative of the final effector mechanisms affecting neurons. Neurodegeneration is indeed substantially absent or very poor in these models. In human patients, on the other side, to investigate CNS myeloid cells is extremely difficult. We think that myeloid extracellular vesicles, representing a “liquid biopsy” of their parental cells, may provide the answers to some of the many question open in this field. Knowing the functional phenotype of myeloid cells during the development of neurological disorders would at least allow to start to investigate their role, protective or damaging, during pathogenic processes. The development of appropriate technologies able to detect, sort, and analyze microvesicles in biological fluids is the necessary pre-requisite. There is considerable pressure to reach these goals, given the implication this would have for relevant fields such as oncology. The sorting and analysis of extracellular vesicles released by neural or CNS-infiltrating cells would be, however, of paramount importance for clinical neurosciences, providing information on an otherwise almost inaccessible organ.

## Conflict of Interest Statement

The authors declare that the research was conducted in the absence of any commercial or financial relationships that could be construed as a potential conflict of interest.
